# Intestinal Dysbiosis in Carriers of Carbapenem-Resistant *Enterobacteriaceae*

**DOI:** 10.1128/mSphere.00173-20

**Published:** 2020-04-29

**Authors:** Hila Korach-Rechtman, Maysaa Hreish, Carmit Fried, Shiran Gerassy-Vainberg, Zaher S. Azzam, Yechezkel Kashi, Gidon Berger

**Affiliations:** a Faculty of Biotechnology and Food Engineering, Technion–Israel Institute of Technology, Haifa, Israel; b Ruth & Bruce Rappaport Faculty of Medicine, Technion–Israel Institute of Technology, Haifa, Israel; c Department of Internal Medicine B, Rambam Health Care Campus, Haifa, Israel; University of Michigan–Ann Arbor

**Keywords:** carbapenem-resistant *Enterobacteriaceae*, CRE, microbiome, intestinal dysbiosis, antibiotic resistance

## Abstract

Infection with carbapenem-resistant *Enterobacteriaceae* (CRE) has become an important challenge in health care settings and a growing concern worldwide. Since infection is preceded by colonization, an understanding of the latter may reduce CRE infections. We aimed to characterize the gut microbiota in CRE carriers, assuming that microbiota alterations precede CRE colonization. We evaluated the gut microbiota using 16S rRNA gene sequencing extracted of fecal samples collected from hospitalized CRE carriers and two control groups, hospitalized noncarriers and healthy adults. The microbiota diversity and composition in CRE-colonized patients differed from those of the control group participants. These CRE carriers displayed lower phylogenetic diversity and dysbiotic microbiota, enriched with members of the family *Enterobacteriaceae*. Concurrent with the enrichment in *Enterobacteriaceae*, a depletion of anaerobic commensals was observed. Additionally, changes in several predicted metabolic pathways were observed for the CRE carriers. Concomitantly, we found higher prevalence of bacteremia in the CRE carriers. Several clinical factors that might induce changes in the microbiota were examined and found to be insignificant between the groups. The compositional and functional changes in the microbiota of CRE-colonized patients are associated with increased risk for systemic infection. Our study results provide justification for attempts to restore the dysbiotic microbiota with probiotics or fecal transplantation.

**IMPORTANCE** The gut microbiota plays important roles in the host’s normal function and health, including protection against colonization by pathogenic bacteria. Alterations in the gut microbial profile can potentially serve as an early diagnostic tool, as well as a therapeutic strategy against colonization by and carriage of harmful bacteria, including antibiotic-resistant pathogens. Here, we show that the microbiota of hospitalized patients demonstrated specific taxa which differed between carriers of carbapenem-resistant *Enterobacteriaceae* (CRE) and noncarriers. The difference in the microbiota also dictates alterations in microbiome-specific metabolic capabilities, in association with increased prevalence of systemic infection. Reintroducing specific strains and/or correction of dysbiosis with probiotics or fecal transplantation may potentially lead to colonization by bacterial taxa responsible for protection against or depletion of antibiotic-resistant pathogens.

## Abstract (precis)

The gut microbiota plays important roles in the host’s normal function and health, including protection against colonization by pathogenic bacteria. Alterations in the gut microbial profile can potentially serve as an early diagnostic tool, as well as a therapeutic strategy against colonization by and carriage of harmful bacteria, including antibiotic-resistant pathogens. Here, we show that the microbiota of hospitalized patients demonstrated specific taxa which differed between carriers of carbapenem-resistant *Enterobacteriaceae* (CRE) and noncarriers. The difference in the microbiota also dictates alterations in microbiome-specific metabolic capabilities, in association with increased prevalence of systemic infection. Reintroducing specific strains and/or correction of dysbiosis with probiotics or fecal transplantation may potentially lead to colonization by bacterial taxa responsible for protection against or depletion of antibiotic-resistant pathogens.

## INTRODUCTION

The emergence and spread of highly antibiotic-resistant bacteria represent a major clinical challenge. In recent years, the numbers of infections caused by bacteria such as Clostridium difficile, methicillin-resistant Staphylococcus aureus, and vancomycin-resistant *Enterococcus* have increased markedly (1). Carbapenem-resistant *Enterobacteriaceae* (CRE) are highly drug-resistant pathogens with a rapidly increasing incidence in a variety of clinical settings (2).

Infections caused by CRE have been associated with increased cost and length of hospital stay as well as frequent treatment failures and death (2). There are several known risk factors (2, 3), including CRE carriage in the gastrointestinal tract (GIT), since this site serves as a source for subsequent clinical infection in approximately 9% of carriers (4). Moreover, CRE carriers serve as a major reservoir for dissemination of these pathogens in health care facilities (4, 5).

The complex commensal microbiota that normally colonizes mucosal surfaces in healthy individuals allows resistance to colonization and inhibits expansion and domination by antibiotic-resistant exogenous bacteria, such as members of the *Enterobacteriaceae* (6, 7). Microbial dysbiosis may lead to an overgrowth of antibiotic-resistant pathogens (8), which can be calamitous for susceptible patients, resulting in bacteremia and sepsis (9), and it is associated with increased risk for transmission due to increased shedding to the environment (10, 11).

It is reasonable to assume that alteration of the normal microbiota may be associated with the development of CRE carriage. Therefore, we aimed to determine the structure of the GIT microbiota in CRE-colonized patients.

## RESULTS

To study the microbiota profile in CRE carriage, we analyzed the clinical parameters and microbial composition of three groups: hospitalized CRE carriers, hospitalized noncarriers, and healthy controls.

### Study cohort clinical characteristics.

The demographic and clinical characteristics of all groups in the study cohort are presented in Tables 1 and 2. There were no significant differences between the groups regarding confounding factors such as gender, ethnic origin, gastrointestinal disease, radiotherapy, chemotherapy, and diabetes mellitus. Moreover, the comparison between these factors and the microbial profile described below was insignificant.

**TABLE 1:** Demographic characteristics of the study cohort

Variable	Result for^*a*^:			*P* value
	Healthy participants (*n* = 15)	Hospitalized non-CRE carriers (*n *= 22)	CRE carriers (*n *= 40)	
Age (yrs)	42.2 ± 3.6 (20–72)	71.1 ± 3.3 (18–88)^*b*^	66.7 ± 2.6 (23–88)^*b*^	**<**0.0001
Gender				
Male	8 (53.3)	13 (59.1)	22 (55)	0.93
Female	7 (46.7)	9 (40.9)	18 (45)	0.93
Ethnic background				
Jewish^*c*^				
Long-term residents	12 (80)	12 (54.6)	16 (40)	0.27
Former Soviet Union immigrants	2 (13.3)	4 (18.2)	12 (30)	0.27
Arabic	1 (6.7)	6 (27.3)	11 (27.5)	0.27
Unknown	0	0	1 (2.5)	0.27

a Ages are given as mean ± standard error of the mean (range); other values are expressed as number (percent) of patients.

b Compared to the healthy group; the difference between the two other study groups was not significant.

c The comparison between immigrants versus long-term residents was conducted in order to eliminate a possible effect on the microbiome.

**TABLE 2:** Clinical characteristics of the hospitalized study cohort

Variable	Result for^*a*^:		*P* value
	CRE carriers (*n* = 40)	Hospitalized non-CRE carriers (*n *= 22)	
GID	8 (20)	1 (4.6)	0.08
Radiotherapy	3 (7.5)	2 (9.1)	NS
Chemotherapy	17 (42.5)	6 (27.3)	0.16
Diabetes mellitus	17 (42.5)	12 (54.6)	NS
Cultures^*b*^ (sputum and/or urine and/or blood)			
Positive	33 (82.5)	16 (72.7)	NS
Negative	4 (10)	5 (22.7)	
Missing	3 (7.5)	1 (4.5)	
Bacteremia			
Positive	19 (47.5)	5 (22.7)	0.03
Negative	17 (42.5)	16 (72.7)	
Missing	4 (10)	1 (4.5)	
Treatment with antibiotics^*c*^			
Yes	34 (85.5)	20 (90.9)	NS
No	2 (5)	1 (4.5)	
NA	4 (10)	1 (4.5)	
Broad-spectrum antibiotics			
All kinds			
Yes	27 (67.5)	17 (77.3)	NS
No	9 (22.5)	4 (18.2)	
NA	4 (10)	1 (4.5)	
Carbapenem			
Yes	10 (25)	3 (13.6)	0.2
No	26 (65)	18 (81.8)	
NA	4 (10)	1 (4.5)	
Narrow-spectrum antibiotics			
Yes	7 (17.5)	3 (13.6)	NS
No	29 (72.5)	18 (81.8)	
NA	4 (10)	1 (4.5)	
Length of stay until recruitment (days)	10 ± 3^*d*^	11 ± 2	NS

a Length of stay is given as mean ± standard error of the mean; other values are expressed as number (percent) of patients. NA, not available.

b Bacteremia caused by any bacteria, and not specifically by CRE.

c All hospitalized participants were receiving antibiotic treatment during the fecal sampling.

d Calculated with exclusion of one patient, who was hospitalized for 263 days.

Hospitalized patients were older than the healthy individuals (average ages, 68.3 and 42.2 years, respectively). In general, antibiotic usage (broad versus narrow spectrum) and positive culture prevalence were similar in both hospitalized groups. However, the rate of bacteremia (i.e., blood infection) was twice as high in the CRE carriers.

Within the carrier group, CRE species included Klebsiella pneumoniae (*n* = 25), *Enterobacter* species (*n* = 4), Escherichia coli (*n* = 4), Citrobacter freundii (*n* = 2), Raoultella ornithinolytica (*n* = 1), and Enterobacter cloacae (*n* = 1). Three samples had missing data; CRE types included only *Klebsiella* carbapenemase (KPC) and OXA48.

Regarding antibiotic usage, it was noted that vancomycin and piperacillin treatments were used more in the CRE carriers, while amikacin and ceftriaxone treatments were used more in the hospitalized noncarriers (Kruskal-Wallis test; *P < *0.05). Most comparative analyses were conducted between the two hospitalized groups (excluding the healthy group), because of the different age average, the strict exclusion criteria, and the lack of antibiotic treatment, which affect the microbiota.

The prevalence of positive bacterial presence in urine, sputum, and blood cultures (not specifically positive for CRE) was around 80% and similar between both hospitalized groups. However, a higher bacteremia rate was found in the CRE carriers than in the noncarriers (53% versus 24%, respectively; *P = *0.03). Interestingly, 74% (14 of 19) of the bacteremias detected in the CRE carriers were caused by *Enterobacteriaceae*, of which 57% were due to Klebsiella pneumoniae. K. pneumoniae bacteremia diversity included one patient with KPC-producing K. pneumoniae, five patients with extended-spectrum beta-lactamase (ESBL)-producing K. pneumoniae, and two with non-carbapenem-resistant K. pneumoniae.

### Microbiota characterization.

To characterize the microbiota, participants’ fecal DNA was subjected to 16S rRNA gene sequencing.

**FIG 1:**
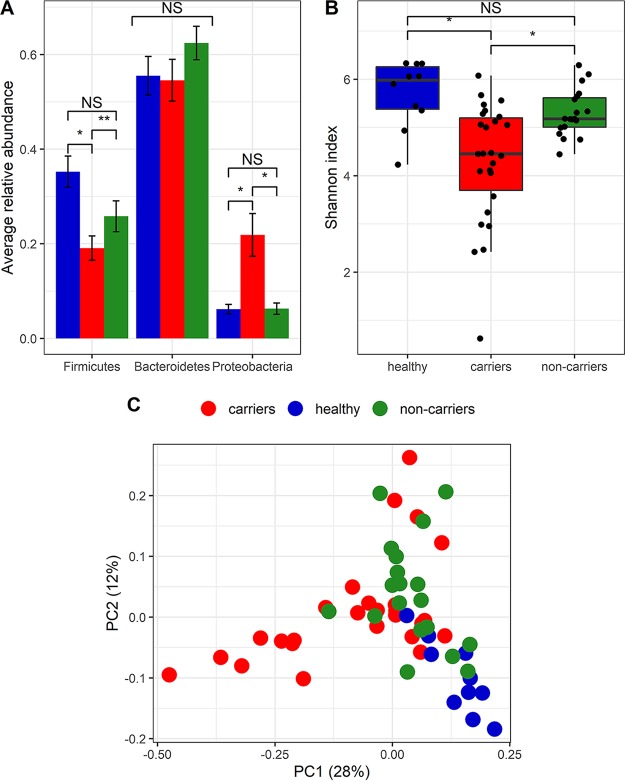
Microbiota composition in the healthy participants, hospitalized CRE carriers, and hospitalized noncarriers. Bacterial composition was assessed by Illumina MiSeq 16S rRNA gene sequencing of fecal DNA samples. (A) Relative abundances of the three dominant phyla in the three experimental groups. Blue, healthy group; red, CRE carriers; green, noncarriers. *, *P < *0.005; **, *P < *0.001; NS, not significant. (B) Alpha diversity between microbial communities was box-plotted based on the Shannon diversity index. (C) Beta diversity between microbial communities was clustered using PCoA based on weighted UniFrac measure.

Taxonomic classification revealed that the dominant bacterial phyla were *Bacteroidetes* (56 to 62%), *Firmicutes* (19 to 35%), and *Proteobacteria* (6 to 21%) (Fig. 1A). *Firmicutes* prevalence was significantly lower in the CRE carriers than in noncarriers and healthy controls (*P < *0.005); numbers of *Proteobacteria* were significantly higher in the CRE carriers (*P < *0.005). The ratio of *Firmicutes* to *Bacteroidetes*, considered highly relevant in human gut microbiota composition (12, 13), was lowest in the CRE carriers (0.35 ± 0.05), higher for hospitalized noncarriers (0.41 ± 0.05), and highest in the healthy group (0.63 ± 0.05) (*P < *0.05).

### Microbial diversity and composition.

Microbial richness assessment, determined using the Shannon index, revealed that CRE carriers had significantly lower richness than the other groups (*P < *0.005) (Fig. 1B). Interestingly, the healthy and noncarrier groups did not differ in the richness measure.

**FIG 2:**
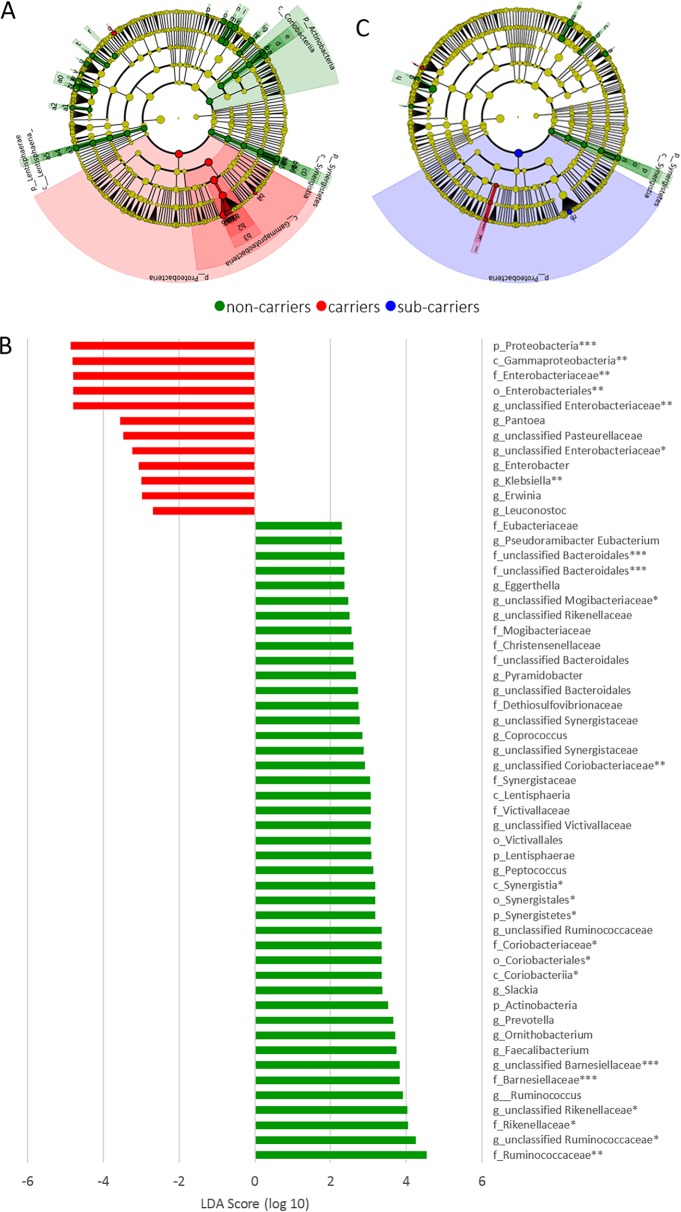
Bacterial markers associated with CRE carriers. Differentially abundant OTUs between CRE carriers (red) and noncarriers (green) were identified using LEfSe and are presented as a cladogram (A) and a histogram of LDA scores (log_10_) (B). Groups are defined according to the microbiota origin. (C) Cladogram of differently abundant OTUs with classes defined according to carriers/noncarriers/subcarriers and subclasses defined according to carriers/noncarriers. Only taxa with an LDA score of ≥2 and a *P* value of <0.05 are shown. *, *P* < 0.01; **, *P* < 0.005; ***, *P* < 0.001.

The bacterial communities of the three groups were compared using principal-coordinate analysis (PCoA) based on weighted UniFrac measure (Fig. 1C). The samples from healthy individuals clustered separately from those from hospitalized participants. The PC1 and PC2 vectors significantly discriminated between the groups (Kruskal-Wallis test; *P < *0.001). We found no significant association between the PCo scores and chemotherapy, radiotherapy, gastrointestinal disease (GID), diabetes mellitus, or other clinical variables. LEfSe (linear discriminant analysis coupled with effect size measures) was used to identify bacterial taxa associated with CRE carriage, by comparing the microbiota of CRE carriers and noncarriers (Fig. 2A). The CRE carriers had a significantly increased prevalence of different genera belonging to the family *Enterobacteriaceae*, including *Pantoea*, *Enterobacter*, *Klebsiella*, and *Erwinia*. Decreased prevalence was observed for the *Rikenellaceae*, *Barnesiellaceae*, and genera belonging to the *Clostridiales*, including *Ruminococcus*, *Faecalibacterium*, *Coprococcus*, and *Ornithobacterium* (see linear discriminative analysis [LDA] scores in Fig. 2C), and anaerobic commensals, with potentially important beneficial roles for the host (6, 7, 14). The decreased abundance of members of the family *Barnesiellaceae* among CRE carriers was also significant in analysis of the microbiotas with the removal of all operational taxonomic units (OTUs) belonging to the family *Enterobacteriaceae*.

Analysis of the bacterial communities by PCoA also enabled detection of a subcluster within the CRE carrier group (Fig. 1C). LEfSe showed that this subcluster had increased prevalence of the phylum *Proteobacteria* and the genus *Klebsiella* (Fig. 2B). This CRE carrier subcluster, as in the CRE carrier group, showed a decreased prevalence of the phylum *Synergistetes*, the family *Barnesiellaceae*, and an unclassified genus of *Barnesiellaceae*. Analysis of the clinical parameters of this subcluster indicated that three of eight patients received ceftriaxone treatment (compared to three of the 32 remaining carriers). However, no demographic or clinical differences between this subcluster and the rest of the CRE carriers were detected. In addition, no significant association was found between the PCo scores of the microbiota and all antibiotic treatments between the hospitalized groups.

We observed significant differences between the microbial communities of the healthy individuals and those of both hospitalized groups (Fig. 1C). Analyses between the healthy group and each of the two hospitalized patient groups separately revealed few taxa characterizing hospitalized patients, including *Klebsiella*, *Enterococcus*, *Eggerthella*, *Citrobacter*, and *Coprobacillus* (see Fig. S1 in the supplemental material). Comparing only the CRE carriers with the healthy group revealed an increase in the abundance of the genera *Enterobacter*, *Klebsiella*, and *Erwinia*, which belong to the family *Enterobacteriaceae*, and decreases in the abundances of *Faecalibacterium* and *Coprococcus*. The prevalence of *Barnesiellaceae* was also decreased in the in CRE carriers compared to the healthy participants.

10.1128/mSphere.00173-20.1 FIG S1 Bacterial markers associated with hospitalization. Differentially abundant operational taxonomic units (OTUs) between healthy participants and noncarriers (left) and between healthy participants and CRE carriers (right) were identified using LEfSe. LDA score bars are colored according to the group in which the bacterial taxon was more abundant (red, CRE carriers; blue, healthy participants; green, noncarriers). Groups are defined according to the microbiota origin. Only taxa with an LDA of ≥2 and a *P* value of <0.05 are shown. Download FIG S1, PDF file, 0.2 MB. Copyright © 2020 Korach-Rechtman et al. 2020 Korach-Rechtman et al. This content is distributed under the terms of the Creative Commons Attribution 4.0 International license.

### Functional prediction of the microbiota.

In this study, we used 16S rRNA genes to study microbial communities in CRE carriage. However, this marker gene cannot directly identify metabolic or other functional capabilities of the microorganisms. Nonetheless, PICRUSt (phylogenetic investigation of communities by reconstruction of unobserved states) is a technique that uses evolutionary modeling to predict metagenomes from 16S data and a reference genome database. PICRUSt-predicted metabolic pathways differ between CRE carriers and noncarriers, and these differing pathways correlated with the relative abundances of the bacteria differentiating the hospitalized groups as identified by LEfSe.

**FIG 3:**
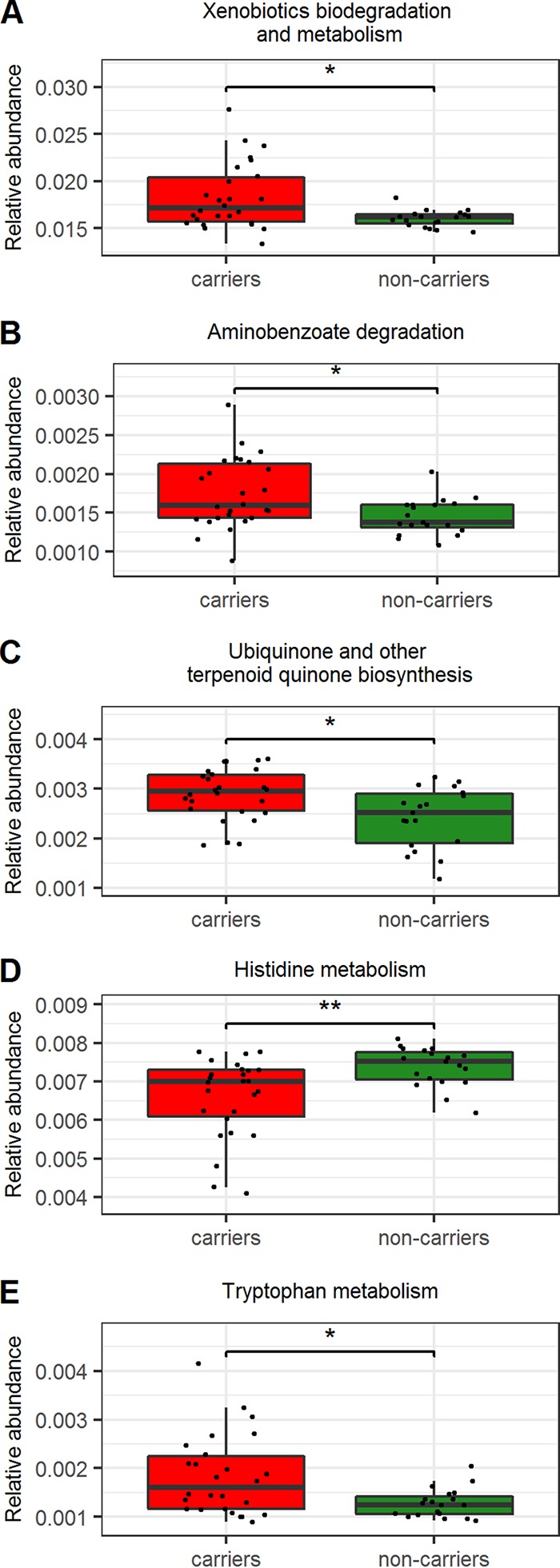
Inferred gut microbiome functions associated with CRE carriage. Predicted microbial functions were inferred by PICRUSt from 16S rRNA gene sequences. The relative abundances of level 2 (A) and level 3 (B to D) KEGG-selected functions of hospitalized CRE carriers and noncarriers are shown. *, *P* < 0.05; **, *P* < 0.005.

We found that CRE carriers were enriched in functional categories associated with xenobiotic biodegradation and metabolism (level 2 [L2]) and amino benzoate degradation (L3) (LDA score = 3.03 and 2.14, respectively; *P < *0.02) (see Fig. S2). Moreover, the family *Enterobacteriaceae* positively correlated with xenobiotic biodegradation and metabolism (*R* = 0.534; false discovery rate [FDR] *P < *0.003). Other functional categories that were different in CRE carriers included reduction in histidine metabolism (L3), elevation in ubiquinone and other terpenoid-quinone biosyntheses (L3), and tryptophan metabolism (L3) (Fig. 3).

10.1128/mSphere.00173-20.2 FIG S2 Inferred gut microbiome functions associated with CRE carriage. Predicted microbial functions of hospitalized CRE carriers and noncarriers were compared by LDA effect size (LEfSe) analysis of KEGG (Kyoto Encyclopedia of Genes and Genomes) based on level 3 (A) and level 2 (B) pathways. Panel C shows the principal-coordinate analysis of selected functions, which were differently expressed between the hospitalized CRE carriers and noncarriers. Download FIG S2, PDF file, 0.3 MB. Copyright © 2020 Korach-Rechtman et al. 2020 Korach-Rechtman et al. This content is distributed under the terms of the Creative Commons Attribution 4.0 International license.

## DISCUSSION

This study has demonstrated that CRE-colonized patients have dysbiotic microbiotas in terms of community membership, with different functional metabolic microbiota profiles.

The intestinal microbiota can protect itself against colonization with new bacteria (colonization resistance), while dysbiosis is apparently exploited by CRE for colonization. On the other hand, it is also possible that the established CRE colonization induces significant perturbations to the microbiota, which in turn may act as a pathogenic community to perpetuate host pathology (15).

We observed that healthy individuals have higher microbial diversity, while CRE carriers have the lowest diversity (Fig. 1B) (16, 17). Moreover, we observed three clusters, indicating different microbial community structures for each of the experimental groups (Fig. 1C). This is in agreement with numerous studies that have shown reduced bacterial diversity in obesity, inflammatory bowel disease, irritable bowel syndrome, and type 2 diabetes mellitus (16, 17). Since CRE carriers and noncarrier hospitalized patients received antibiotic treatments, the enrichment found in *Klebsiella*, *Enterococcus*, and *Citrobacter* compared to the healthy group supports a previous study indicating that antibiotic treatment promotes intestinal colonization by *Enterococcus* and *Enterobacteriaceae* (8). Few specific taxa of the microbiota were different between the CRE carriers and noncarriers. First, in addition to the CRE themselves, we observed increased abundance of *Enterobacteriaceae* (*Enterobacter*, *Erwinia*, *Pantoea*, and *Klebsiella*), among which were resident species with virulence potential that are normally kept at low levels. This consequently predisposes the host to infections with life-threatening sequelae caused by the CRE themselves and potentially other pathobionts.

Second, concurrently with the enrichment in *Enterobacteriaceae*, a depletion of anaerobic commensals was observed (Fig. 2), among which were *Coprococcus* and *Faecalibacterium*, two important short-chain fatty acid (SCFA)-producing commensal bacteria (14). These SCFAs are physiological by-products of carbohydrate fermentation by the microbiota and serve to salvage energy for the host, enhance the mucosal barrier, and inhibit intestinal inflammation and oxidative stress (18). Among the functional consequences of reduction in anaerobic bacteria is a reduced metabolic capacity, often exemplified by a decline in SCFA production. Dysbiosis caused by broad-spectrum antibiotics (e.g., clindamycin and cephalosporins), which can presumably enable CRE colonization, is commonly associated with low intestinal SCFA levels (18).

Colonization resistance depends on microbiota diversity, as well as microbial composition. The intestinal microbiota can protect efficiently against colonization by many enteric pathogens (7, 19, 20). Therefore, it is not surprising that during dysbiosis, intestinal colonization resistance is impaired. *Barnesiella* spp. were less abundant in CRE carriers than in the noncarriers and the healthy control group. Interestingly, it was shown that *Barnesiella* spp. have the ability to restrict the growth of intestinal pathogens and limit colonization with highly antibiotic-resistant bacteria, and they are required to prevent expansion of oxygen-tolerant bacteria such as *Enterobacteriaceae* (6, 7, 20).

As an outcome of dysbiosis, predictions of metabolic function also indicated a profile shift. In CRE carriers, we found changes in the abundances of several pathways, including increased histidine metabolism and decreased biosynthesis of ubiquinone and other terpenoid quinones, tryptophan metabolism, xenobiotic biodegradation and metabolism, and amino benzoate degradation (Fig. 3). These metabolic alterations were previously linked to modulation of the immune system response to pathogens and the adaptive immune system activation (21, 22) and to compromised intestinal epithelial barrier and function, which allow bacterial translocation (6, 23).

The xenobiotic biodegradation and metabolism pathway, and specifically the aminobenzoate degradation pathway, generates catechol (1,2-dihydroxybenzene), which promotes *Enterobacteriaceae* growth and virulence (24). This can explain the enrichment of this pathway (Fig. 3) and its positive correlation with *Enterobacteriaceae* and may suggest a causative scenario in which the change in the microbiota leads to *Enterobacteriaceae* enrichment. Taken together, the functional prediction of the microbiota leading to enrichment in *Enterobacteriaceae*, the immune system modulation, and the intestinal epithelial damage can explain the higher rate of bloodstream infections in the CRE carriers, since colonization with *Enterobacteriaceae* has been associated with increased risk for bacteremia (25).

These compositional and functional changes predispose the host to invasive infection and death. We found higher rates of bacteremia (not caused only by CRE) in the CRE carrier group than in the noncarriers (Table 2). Interestingly, only one patient had bacteremia with KPC-producing K. pneumoniae, which is consistent with a previous study showing that K. pneumoniae isolates from blood samples were less likely to harbor KPC (3). This can be explained by the fitness costs of resistance, typically observed as a reduced bacterial growth rate (26).

On the basis of other studies, we speculate that a dysbiotic microbiota and a high rate of bacteremia in CRE carriers are linked by low levels of SCFAs. SCFAs were shown to interact with innate mechanisms of defense against infection (regulation of immune cell function) and low levels of “defensive bacteria,” such as *Barnesiella* (6, 18).

Based on this study, it is not possible to determine causality between dysbiosis and CRE colonization, as dysbiosis can be both a cause and a result of CRE colonization. One major limitation of our study is the strong impact of antibiotic treatment on the gut microbiota (27). To weaken the effect, one of our control groups was composed of hospitalized non-CRE carriers. Both hospitalized groups were hospitalized (for at least 7 days) at the same health care facility and were treated with an antibiotic profile similar to that of the hospitalized CRE carriers. As mentioned, no significant confounding factors differed between the CRE carriers and noncarriers, except for the CRE carriage itself. There may be other unexamined factors that may cause the microbial differences we found. Moreover, all taxon-associated analyses were conducted by excluding the healthy (not hospitalized) controls, since they were not specifically tested for CRE carriage and the lack of antibiotic treatment does not allow proper comparison between the groups.

Once established, the gut microbiota composition is relatively stable throughout adult life, but it can alter as a result of the action of several vectors. In our study, a trend toward a statistically significant difference between the experimental groups was found in the following factors: treatment with carbapenem, chemotherapy treatment, and gastrointestinal disease or disorder. However, we cannot point to the exact determinants influencing the microbiota composition in CRE carriers. Whatever the predominant factors that modify the microbiota are, the result is an “unhealthy microbiota” which has lost key species required for shaping a “healthy microbiota.” Indeed, the gut microbiota has been previously shown to affect susceptibility to infections caused by other pathogens, such as Vibrio cholerae (28) and C. difficile (29).

### Conclusions.

Overall, the results in our cohort indicate that the interrelation between dysbiotic microbiota, its pool of bacterial genes (microbiome), and their expressed functions might weaken the protection and resistance against colonization and infection with CRE and other pathobionts. Therefore, our study supports the challenging possibility of fecal transplantation as a therapeutic strategy for CRE carriage, a strategy already efficiently used to treat recurrent C. difficile infection (18, 30) and studied for eradication of carriage of other highly drug-resistant enteric bacteria (7, 31, 32). Reintroducing specific strains and/or correction of dysbiosis with probiotics or fecal transplantation may potentially lead to restoration of colonization resistance (7, 31).

## MATERIALS AND METHODS

### Study design and participants.

The study population was composed of two control groups (15 healthy adults and 22 hospitalized non-CRE carriers) and one group of CRE carriers (*n *= 40). Hospitalized adults were recruited from the Division of Internal Medicine at Rambam Health Care Campus (Haifa, Israel); nonhospitalized healthy participants were recruited from the local community and most likely were not CRE carriers. Within the hospitalized groups, CRE carriage was determined upon hospitalization and every other week by routine screening for rectal carriage by PCR of five genes (KPC, NDM, OXA48, VIM, and IMI). The average hospitalization periods prior to recruitment and sample collection were 10 and 11 days (carriers and noncarriers, respectively), with the exclusion of one CRE carrier patient, who was hospitalized for 263 days.

The following exclusion criteria were applied to the healthy group to avoid factors capable of altering the microbiome: current smoker, active or recent (within 6 months) chemotherapy and/or radiation treatment, homeopathic-preparation use, current infectious disease, chronic or acute gastrointestinal tract disease (including Clostridium difficile infection), and antibiotic treatment or vaccination within the last 6 months prior to sample collection. This group of individuals volunteered and were recruited randomly by hospital staff. Nonetheless, due to the lack of antibiotic treatment, the differences in participants’ average ages, and the fact they were not hospitalized under similar conditions, the majority of comparative analyses were conducted between the two hospitalized groups. Inclusion criteria for CRE carriers included hospitalization following CRE identification conducted upon hospitalization. Inclusion criteria for hospitalized noncarriers included hospitalization for at least 6 days, treatment with antibiotics, and negative tests for carriage of any antibiotic-resistant bacteria.

Written informed consent was obtained from all study participants. The study was approved by the Rambam Health Care Campus ethics committee (approval number 0418-14-RMB). This study conformed to the Helsinki Declaration and to local legislation.

Clinical analyses were performed on the entire study population (*n *= 77), and 55 samples from the three groups were sent for sequencing (the earliest recruits; 10 healthy participants, 19 noncarriers, and 26 carriers). Clinical variables, including medical background, and laboratory parameters, were retrieved from medical records (Tables 1 and 2).

### Sample collection.

Fresh fecal samples were collected from hospitalized participants by the research cadre (CRE carrier and noncarrier groups) immediately after recruitment. Following collection, swabs were stored at −80ºC. Samples from healthy participants were self-collected by participants, transported in a freezer pack to the laboratory within 24 h of collection, and then stored at −80ºC.

### Microbiota sequencing and taxonomy assignment.

Total DNA was extracted and amplified as previously described (33, 42). Briefly, total fecal DNA was extracted using a QIAamp DNA stool minikit (Qiagen, Hilden, Germany). PCR amplification of the 16S rRNA gene V3-V4 region was conducted using primers CS1-341F and CS2-806R (34). Samples were sequenced at the DNA Services Facility, University of Illinois at Chicago, using a dual PCR strategy (35). Samples were barcoded in a second PCR on an Illumina MiSeq sequencer, using standard V3 chemistry with paired-end 300-bp reads. The resulting paired-end FASTQ files were merged using the PEAR software package. Primer sequence removal and length trimming (for sequences of <390 bp) were conducted using the software package CLC Genomics Workbench (v7; CLC Bio, Qiagen, Boston, MA). Sequences were screened for chimeras using the usearch61 algorithm (36), and putative chimeras were removed from the data set. Sequence data were processed using the Quantitative Insight into Microbial Ecology (QIIME) 1.8.0 pipeline. Operational taxonomic units (OTUs) were defined based on 97% similarity clustering using the UCLUST algorithm (37). Taxonomy was assigned against the Greengenes database (v13_8) as the reference (38).

### Microbiota composition and metabolic analysis.

Diversity analyses were calculated with a rarefied OTU table containing 40,000 reads per sample and were conducted twice, i.e., with and without taxa belonging to the family *Enterobacteriaceae*. Alpha diversity was calculated using the Shannon diversity index and visualized by box plots using the statistical software environment R.

Beta diversity was determined by computing weighted UniFrac distance, and the resulting matrices were visualized by principal-coordinate analysis (PCoA) plots. The PCo score comparison based on the Kruskal-Wallis test was used to analyze the association between community composition among the study groups and the clinical variables using R.

Linear discriminant analysis coupled with effect size measures (LEfSe) (39) was used to identify the taxa differentiating the experimental groups (healthy participants versus CRE carriers; CRE carriers versus noncarriers; healthy participants versus noncarriers; hospitalized bacteremia patients versus nonbacteremia patients). Taxa showing an LDA score of <2.5 were further compared and correlated with all the clinical variables, including CRE type and species, and with the different antibiotic treatments by the Kruskal-Wallis test using R. The antibiotic treatment was also tested by combining treatment targets (i.e., Gram-positive bacteria, Gram-negative bacteria, broad-spectrum antibiotic).

Bacterial metabolic activity abundance, as defined by the Kyoto Encyclopedia of Genes and Genomes (KEGG) (40), was generated by PICRUSt (version 1.1.3) (41). PICRUSt imputes metabolic pathways from 16S data only for taxa with the entire genome available as a reference. It was implemented using closed reference OTUs picked by QIIME over the same set of sequences. LEfSe was used to identify functional attributes differentiating between CRE carriers and noncarriers. Selected functional attributes were compared based on the Kruskal-Wallis test using R. Relative abundance of bacteria with an LDA score of >2 in the LEfSe analyses between hospitalized CRE carriers and noncarriers was correlated with level 2 and 3 (L2 and L3) functional profiles. Pearson correlation coefficients and FDR-corrected *P* values were calculated using R.

## References

[1] Snitkin ES, Zelazny AM, Thomas PJ, Stock F, Henderson DK, Palmore TN, Segre JA 2012 Tracking a hospital outbreak of carbapenem-resistant Klebsiella pneumoniae with whole-genome sequencing. Sci Transl Med 4:148ra116. doi:10.1126/scitranslmed.3004129. PMC3521604 22914622

[2] Gupta N, Limbago BM, Patel JB, Kallen AJ 2011 Carbapenem-resistant Enterobacteriaceae: epidemiology and prevention. Clin Infect Dis 53:60–67. doi:10.1093/cid/cir202. 21653305

[3] Gasink LB, Edelstein PH, Lautenbach E, Synnestvedt M, Fishman NO 2009 Risk factors and clinical impact of Klebsiella pneumoniae carbapenemase-producing K. pneumoniae. Infect Control Hosp Epidemiol 30:1180–1185. doi:10.1086/648451. 19860564 PMC2893218

[4] Campos AC, Albiero J, Ecker AB, Kuroda CM, Meirelles LEF, Polato A, Tognim MCB, Wingeter MA, Teixeira J 2016 Outbreak of Klebsiella pneumoniae carbapenemase–producing K pneumoniae: a systematic review. Am J Infect Control 44:1374–1380. doi:10.1016/j.ajic.2016.03.022. 27156198

[5] Bilavsky E, Schwaber MJ, Carmeli Y 2010 How to stem the tide of carbapenemase-producing Enterobacteriaceae?: proactive versus reactive strategies. Curr Opin Infect Dis 23:327–331. doi:10.1097/QCO.0b013e32833b3571. 20581673

[6] Montassier E, Al-Ghalith GA, Ward T, Corvec S, Gastinne T, Potel G, Moreau P, De La Cochetiere MF, Batard E, Knights D 2016 Pretreatment gut microbiome predicts chemotherapy-related bloodstream infection. Genome Med 8:49. doi:10.1186/s13073-016-0301-4. 27121964 PMC4848771

[7] Caballero S, Kim S, Carter RA, Leiner IM, Sušac B, Miller L, Kim GJ, Ling L, Pamer EG 2017 Cooperating commensals restore colonization resistance to vancomycin-resistant Enterococcus faecium. Cell Host Microbe 21:592–602.E4. doi:10.1016/j.chom.2017.04.002. 28494240 PMC5494988

[8] Ubeda C, Taur Y, Jenq RR, Equinda MJ, Son T, Samstein M, Viale A, Socci ND, Van Den Brink MRM, Kamboj M, Pamer EG 2010 Vancomycin-resistant Enterococcus domination of intestinal microbiota is enabled by antibiotic treatment in mice and precedes bloodstream invasion in humans. J Clin Invest 120:4332–4341. doi:10.1172/JCI43918. 21099116 PMC2993598

[9] Taur Y, Xavier JB, Lipuma L, Ubeda C, Goldberg J, Gobourne A, Lee YJ, Dubin KA, Socci ND, Viale A, Perales M-A, Jenq RR, Van Den Brink MRM, Pamer EG 2012 Intestinal domination and the risk of bacteremia in patients undergoing allogeneic hematopoietic stem cell transplantation. Clin Infect Dis 55:905–914. doi:10.1093/cid/cis580. 22718773 PMC3657523

[10] Livorsi DJ, Arif S, Garry P, Kundu MG, Satola SW, Davis TH, Batteiger B, Kressel AB 2015 Methicillin-resistant Staphylococcus aureus (MRSA) nasal real-time PCR: a predictive tool for contamination of the hospital environment. Infect Control Hosp Epidemiol 36:34–39. doi:10.1017/ice.2014.16. 25627759 PMC4793965

[11] Donskey CJ, Chowdhry TK, Hecker MT, Hoyen CK, Hanrahan JA, Hujer AM, Hutton-Thomas RA, Whalen CC, Bonomo RA, Rice LB 2000 Effect of antibiotic therapy on the density of vancomycin-resistant enterococci in the stool of colonized patients. N Engl J Med 343:1925–1932. doi:10.1056/NEJM200012283432604. 11136263 PMC4370337

[12] Mariat D, Firmesse O, Levenez F, Guimarăes V, Sokol H, Doré J, Corthier G, Furet J-P 2009 The Firmicutes/Bacteroidetes ratio of the human microbiota changes with age. BMC Microbiol 9:123. doi:10.1186/1471-2180-9-123. 19508720 PMC2702274

[13] Ley RE, Turnbaugh PJ, Klein S, Gordon JI 2006 Microbial ecology: human gut microbes associated with obesity. Nature 444:1022–1023. doi:10.1038/4441022a. 17183309

[14] Ríos-Covián D, Ruas-Madiedo P, Margolles A, Gueimonde M, de Los Reyes-Gavilán CG, Salazar N 2016 Intestinal short chain fatty acids and their link with diet and human health. Front Microbiol 7:185. doi:10.3389/fmicb.2016.00185. 26925050 PMC4756104

[15] Shen P, Whelan FJ, Schenck LP, McGrath JJC, Vanderstocken G, Bowdish DME, Surette MG, Stämpfli MR 2017 Streptococcus pneumoniae colonization is required to alter the nasal microbiota in cigarette smokeexposed mice. Infect Immun 85:e00434-17. doi:10.1128/IAI.00434-17. PMC5607400 28760931

[16] Integrative HMP Research Network Consortium. 2014 The Integrative Human Microbiome Project: dynamic analysis of microbiome-host omics profiles during periods of human health and disease. Cell Host Microbe 16:276–289. doi:10.1016/j.chom.2014.08.014. 25211071 PMC5109542

[17] Sommer F, Anderson JM, Bharti R, Raes J, Rosenstiel P 2017 The resilience of the intestinal microbiota influences health and disease. Nat Rev Microbiol 15:630–638. doi:10.1038/nrmicro.2017.58. 28626231

[18] Maslowski KM, Mackay CR 2011 Diet, gut microbiota and and immune responses. Nat Immunol 12:5–9. doi:10.1038/ni0111-5. 21169997

[19] Gosalbes MJ, Vázquez-Castellanos JF, Angebault C, Woerther P-L, Ruppé E, Ferrús ML, Latorre A, Andremont A, Moya A 2016 Carriage of Enterobacteria producing extended-spectrum β-lactamases and composition of the gut microbiota in an Amerindian community. Antimicrob Agents Chemother 60:507–514. doi:10.1128/AAC.01528-15. 26552974 PMC4704183

[20] Ubeda C, Bucci V, Caballero S, Djukovic A, Toussaint NC, Equinda M, Lipuma L, Ling L, Gobourne A, No D, Taur Y, Jenq RR, van den Brink MRM, Xavier JB, Pamer EG 2013 Intestinal microbiota containing Barnesiella species cures vancomycin-resistant Enterococcus faecium colonization. Infect Immun 81:965–973. doi:10.1128/IAI.01197-12. 23319552 PMC3584866

[21] Jiang X, Chen ZJ 2012 The role of ubiquitylation in immune defence and pathogen evasion. Nat Rev Immunol 12:35–48. doi:10.1038/nri3111. PMC3864900 22158412

[22] Ashida H, Kim M, Sasakawa C 2014 Exploitation of the host ubiquitin system by human bacterial pathogens. Nat Rev Microbiol 12:399–413. doi:10.1038/nrmicro3259. 24801936

[23] Dinh DM, Volpe GE, Duffalo C, Bhalchandra S, Tai AK, Kane AV, Wanke CA, Ward HD 2015 Intestinal microbiota, microbial translocation, and systemic inflammation in chronic HIV infection. J Infect Dis 211:19–27. doi:10.1093/infdis/jiu409. 25057045 PMC4326316

[24] Rooks MG, Veiga P, Wardwell-Scott LH, Tickle T, Segata N, Michaud M, Gallini CA, Beal C, Van Hylckama-Vlieg JE, Ballal SA, Morgan XC, Glickman JN, Gevers D, Huttenhower C, Garrett WS 2014 Gut microbiome composition and function in experimental colitis during active disease and treatment-induced remission. ISME J 8:1403–1417. doi:10.1038/ismej.2014.3. 24500617 PMC4069400

[25] Ruppé E, Andremont A 2013 Causes, consequences, and perspectives in the variations of intestinal density of colonization of multidrug-resistant enterobacteria. Front Microbiol 4:129. doi:10.3389/fmicb.2013.00129. 23755045 PMC3664761

[26] Andersson DI, Hughes D 2010 Antibiotic resistance and its cost: is it possible to reverse resistance? Nat Rev Microbiol 8:260–271. doi:10.1038/nrmicro2319. 20208551

[27] Jeffery IB, Lynch DB, O'Toole PW 2016 Composition and temporal stability of the gut microbiota in older persons. ISME J 10:170–113. doi:10.1038/ismej.2015.88. 26090993 PMC4681863

[28] Weil A, Midani F, Chowdhury F, Khan A, Begum Y, Charles R, Calderwood SB, Ryan ET, Harris J, Qadri F, David L, Larocque R 2015 The gut microbiome and susceptibility to Vibrio cholerae infection. Open Forum Infect Dis 2(Suppl 1):697. doi:10.1093/ofid/ofv131.72.

[29] Theriot CM, Koenigsknecht MJ, Carlson PE, Hatton GE, Nelson AM, Li B, Huffnagle GB, Li JZ, Young VB 2014 Antibiotic-induced shifts in the mouse gut microbiome and metabolome increase susceptibility to Clostridium difficile infection. Nat Commun 5:3114. doi:10.1038/ncomms4114. 24445449 PMC3950275

[30] Borody TJ, Khoruts A 2011 Fecal microbiota transplantation and emerging applications. Nat Rev Gastroenterol Hepatol 9:88–96. doi:10.1038/nrgastro.2011.244. 22183182

[31] Manges AR, Steiner TS, Wright AJ 2016 Fecal microbiota transplantation for the intestinal decolonization of extensively antimicrobial-resistant opportunistic pathogens: a review. Infect Dis (Lond) 48:587–592. doi:10.1080/23744235.2016.1177199. 27194400

[32] Grosen AK, Karmisholt Grosen A, Povlsen JV, Lemming LE, Mark S, Jørgensen D, Dahlerup JF, Hvas CL 2019 Faecal microbiota transplantation eradicated extended-spectrum beta-lactamase-producing Klebsiella pneumoniae from a renal transplant recipient with recurrent urinary tract infections. Case Rep Nephrol Dial 9:102–107. doi:10.1159/000502336. 31559265 PMC6751418

[33] Korach-Rechtman H, Freilich S, Gerassy-Vainberg S, Buhnik-Rosenblau K, Danin-Poleg Y, Bar H, Kashi Y 2019 Murine genetic background has a stronger impact on the composition of the gut microbiota than maternal inoculation or exposure to unlike exogenous microbiota. Appl Environ Microbiol 85:e00826-19. doi:10.1128/AEM.00826-19. 31350316 PMC6715835

[34] Rom O, Korach-Rechtman H, Hayek T, Danin-Poleg Y, Bar H, Kashi Y, Aviram M 2017 Acrolein increases macrophage atherogenicity in association with gut microbiota remodeling in atherosclerotic mice: protective role for the polyphenol-rich pomegranate juice. Arch Toxicol 91:1709–1725. doi:10.1007/s00204-016-1859-8. 27696135

[35] Green SJ, Venkatramanan R, Naqib A 2015 Deconstructing the polymerase chain reaction: understanding and correcting bias associated with primer degeneracies and primer-template mismatches. PLoS One 10:e0128122. doi:10.1371/journal.pone.0128122. 25996930 PMC4440812

[36] Edgar RC 2010 Search and clustering orders of magnitude faster than BLAST. Bioinformatics 26:2460–2461. doi:10.1093/bioinformatics/btq461. 20709691

[37] Caporaso JG, Kuczynski J, Stombaugh J, Bittinger K, Bushman FD, Costello EK, Fierer N, Pena AG, Goodrich JK, Gordon JI, Huttley GA, Kelley ST, Knights D, Koenig JE, Ley RE, Lozupone CA, McDonald D, Muegge BD, Pirrung M, Reeder J, Sevinsky JR, Turnbaugh PJ, Walters WA, Widmann J, Yatsunenko T, Zaneveld J, Knight R 2010 QIIME allows analysis of high-throughput community sequencing data. Nat Methods 7:335–336. doi:10.1038/nmeth.f.303. 20383131 PMC3156573

[38] McDonald D, Price MN, Goodrich J, Nawrocki EP, DeSantis TZ, Probst A, Andersen GL, Knight R, Hugenholtz P 2012 An improved Greengenes taxonomy with explicit ranks for ecological and evolutionary analyses of bacteria and archaea. ISME J 6:610–618. doi:10.1038/ismej.2011.139. 22134646 PMC3280142

[39] Segata N, Izard J, Waldron L, Gevers D, Miropolsky L, Garrett WS, Huttenhower C 2011 Metagenomic biomarker discovery and explanation. Genome Biol 12:R60. doi:10.1186/gb-2011-12-6-r60. 21702898 PMC3218848

[40] Kanehisa M, Goto S 2000 KEGG: Kyoto encyclopedia of genes and genomes. Nucleic Acids Res 28:27–30. doi:10.1093/nar/28.1.27. 10592173 PMC102409

[41] Langille MGI, Zaneveld J, Gregory Caporaso J, McDonald D, Knights D, Reyes JA, Clemente JC, Burkepile DE, Vega Thurber RL, Knight R, Beiko RG, Huttenhower C 2013 Predictive functional profiling of microbial communities using 16S rRNA marker gene sequences. Nat Biotechnol 31:814–821. doi:10.1038/nbt.2676. 23975157 PMC3819121

[42] Gerassy-Vainberg S, Blatt A, Danin-Poleg Y, Gershovich K, Sabo E, Nevelsky A, Daniel S, Dahan A, Ziv O, Dheer R, Abreu MT, Koren O, Kashi Y, Chowers Y 2018 Radiation induces proinflammatory dysbiosis: transmission of inflammatory susceptibility by host cytokine induction. Gut 67:97–107. doi:10.1136/gutjnl-2017-313789. 28438965

