# Supplementary figures and images for: Intestinal Dysbiosis in Carriers of Carbapenem-Resistant Enterobacteriaceae
Source: mSphere. 2020 Apr 29;5(2):e00173-20. doi: 10.1128/mSphere.00173-20 (PMC7193040; doi:10.1128/mSphere.00173-20)

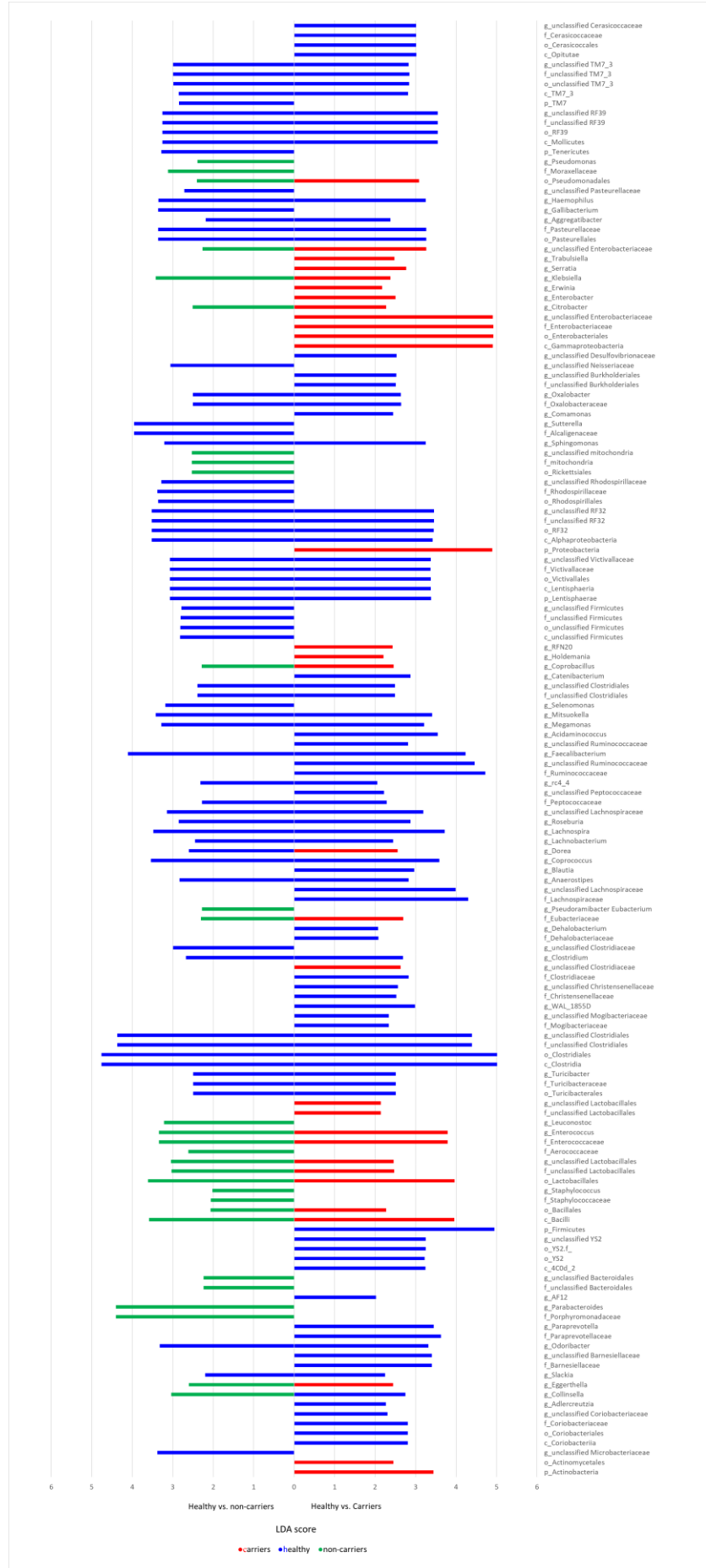

Supplement: FIG S1 [file mSphere.00173-20-sf001.pdf]

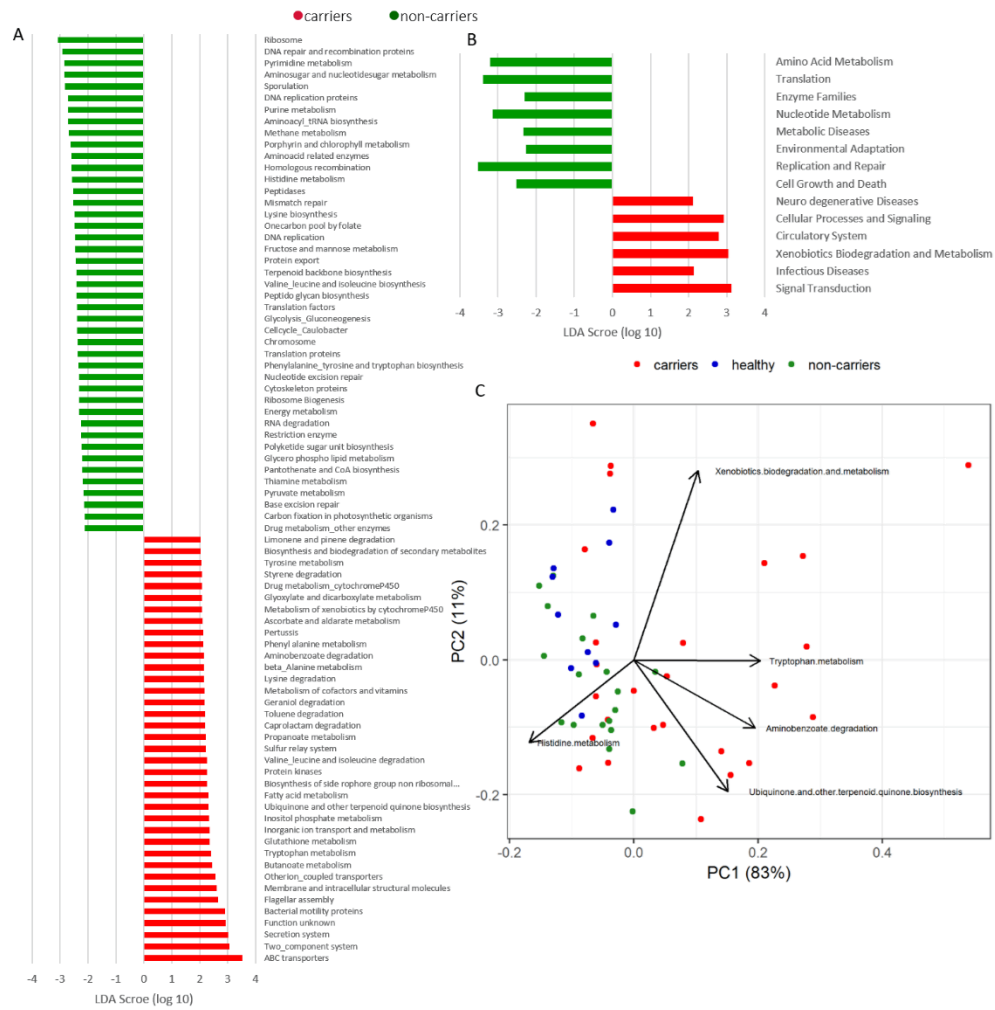

Supplement: FIG S2 [file mSphere.00173-20-sf002.pdf]
